# The effects of prior high intensity double poling on subsequent diagonal stride skiing characteristics

**DOI:** 10.1186/s40064-015-0796-y

**Published:** 2015-02-01

**Authors:** Glenn Björklund, Hans-Christer Holmberg, Thomas Stöggl

**Affiliations:** Department of Health Sciences, Swedish Winter Sports Research Centre, Mid-Sweden University, 831 25 Östersund, Sweden; Swedish Olympic Committee, Stockholm, Sweden; Department of Sport Science and Kinesiology, University of Salzburg, Salzburg, Austria

**Keywords:** EMG, Force, Oxygen extraction, Oxygen uptake

## Abstract

**Purpose:**

To investigate the influence of prior high intensity double poling (DP) on physiological and biomechanical responses during subsequent diagonal stride (DIA).

**Methods:**

Eight well-trained male cross-country skiers (age 22 ± 3 yr; VO_2max_ 69 ± 3 ml · kg^−1^ · min^−1^) roller-skied on a treadmill sequentially for 3 min at 90% DIA VO_2max_ (DIA_1_), 3 min at 90% DP VO_2peak_ and 3 min at 90% DIA VO_2max_ (DIA_2_). Cardio-respiratory responses were monitored continuously and gases and metabolites in blood from the *a. femoralis, v. femoralis* and *v. subclavia* determined. Pole and plantar forces and EMG from 6 lower- and upper-body muscles were measured.

**Results:**

VO_2_ decreased from DIA_1_ to DP and increased again to DIA_2_ (both *P* < 0.05), with no difference between the DIA sessions. Blood lactate rose from DIA_1_ to DP to DIA_2_. O_2_ extraction was attenuated during DP (*P* < 0.05), but was the same during DIA_1_ and DIA_2_. EMG_RMS_ for arm muscles during poling phase, as well as peak pole force and cycle rate were higher, while leg muscle activity was lower during DP than both sessions of DIA (all *P* < 0.05). The ratio of upper-/whole-body EMG_RMS_ correlated negatively with O_2_ extraction in the arms during both sessions of DIA (*P* < 0.05).

**Conclusions:**

In well-trained skiers skiing at high-intensity DP prior to DIA did not influence VO_2_, muscle activation or forces in the latter. At race intensity DP does not influence the distribution of work between upper- and lower-body during a subsequent bout of DIA. O_2_ extraction is coupled to technical skills during skiing.

## Background

Of the several different sub-techniques involved in classical cross-country skiing, diagonal stride (DIA) and double poling (DP) have been most frequently studied. With DIA, the skier utilizes both the arms and legs in a diagonal fashion for propulsion and this is considered to be whole-body exercise (Lindinger et al. 2009). In the case of DP, most propulsive force is supplied by the upper-body, although a dynamic leg movement also contributes to the application of body weight to pole forces (Holmberg et al. 2006; Rud et al. 2014). Consequently, in connection with both of these sub-techniques, and particularly DP, recovery of the upper-body muscles between strokes might be limited.

With regards to cardiovascular regulation, with a similar absolute pulmonary oxygen uptake (VO_2_), delivery of O_2_ to the arms and VO_2_ are higher, but extraction of O_2_ by the arms lower with DP than DIA (Calbet et al. 2005). In the case of DIA, when exercise intensity is reduced from high to moderate, O_2_ extraction in the arms is lowered to a greater extent than in the legs, probably due to a more pronounced decrease in the activation of arm muscles (Björklund et al. 2010). With DP the opposite is observed, i.e., O_2_ extraction in the arms is reduced to a lesser extent than in the legs as the intensity of exercise is diminished, apparently because strong activation of the muscles of the upper-body is maintained (Stöggl et al. 2013).

Furthermore, the skeletal muscles of the arms and legs differ with respect to the production and oxidation of lactate, with the arms producing more at a given workload (Ahlborg and Jensen-Urstad 1991). Thus, more extensive upper-body activation during DP compared to DIA should lead to a higher systemic concentration of lactate with the former sub-technique, as has, indeed, been shown by Van Hall et al. (2003) to be the case with exercise of moderate intensity (~76% VO_2max_). In that investigation and the study by Calbet et al. (2005), the cardiovascular and metabolic influence of DP on subsequent DIA were analyzed to a certain extent, but no direct comparisons between DIA before and after DP were made. Moreover, analysis of higher exercise intensities (race intensity) is also important, since the distinctly higher muscle activation and application of force involved might cause mechanical hindrance of O_2_ extraction and thereby elevate blood levels of lactate (Stöggl et al. 2013).

Whereas DIA and DP have both been characterized individually from a physiological and biomechanical perspective during simulated races (Larsson and Henriksson-Larsen 2005; Mognoni et al. 2001; Mygind et al. 1994; Ortenblad et al. 2011; Welde et al. 2003), the influence of these sub-techniques on one another remains to be examined. Previous investigations on different modes of exercises involving the legs have revealed that the biomechanical and physiological characteristics of running are influenced negatively by prior cycling in the case of moderately trained, but not elite triathletes (Bonacci et al. 2010, 2011). In addition, after this transition, only the latter preserve their running economy, with no change in VO_2_. With repeated sprints of cross-country skiing the fatigue experienced by the upper-body muscles already during the first sprint leads to a shift in body position and, consequently, less effective application of force during DP (Zory et al. 2009, 2011).

Accordingly, the upper-body muscle fatigue induced by high-intensity DP might alter the relative activation of arm and leg muscles, together with pole and plantar forces, during subsequent high-intensity DIA. Thus, our aim here was to examine the influence of DP on subsequent DIA at a simulated race intensity of ~90% of VO_2max/peak_ from a biomechanical and physiological perspective. The hypothesis was that DP alters the distribution of work between the upper- and lower-body, i.e., decreases pole force and activation of arm muscles during subsequent DIA, thereby reducing the O_2_ extraction by the arms.

## Methods

### Subjects

The 8 well-trained male cross-country skiers who volunteered (Table 1) were informed about the test procedures and possible risks prior to providing their written consent to participate. The research techniques and experimental protocol were pre-approved by the Regional Ethical Review Board in Umeå, Sweden (#08-049M) and performed in accordance with the Declaration of Helsinki.

**Table 1:** **Anthropometric and physiological characteristics of the 8 well-trained male cross-country skiers who participated**

**Variable**	**Mean ± SD**
Age (yr)	22 ± 3
Height (cm)	183 ± 4
Weight (kg)	79 ± 6
BMI	23.6 ± 2.1
VO_2max_ (l · min^−1^)	5.4 ± 0.3
VO_2max_ (ml · kg^−1^ · min^−1^)	69 ± 3
VO_2peak_ (l · min^−1^)	4.8 ± 0.4
VO_2peak_ (ml · kg^−1^ · min^−1^)	62 ± 3
OBLA (%VO_2max_)	82 ± 4
OBLA (l · min^−1^)	4.4 ± 0.4
LT (%VO_2max_)	76 ± 3
LT (l · min^−1^)	4.1 ± 0.4
HR_max_ (beats · min^−1^)	189 ± 5

VO_2max_, Maximal O_2_ uptake; OBLA, onset of blood lactate accumulation (4 mmol⋅l^−1^); LT, Lactate threshold; HR_max_, Maximal heart rate.

### The general experimental protocol

All tests were performed on a motor-driven treadmill (Rodby RL 3000, Vänge, Sweden) and, to minimize variations in rolling resistance, using the same pair of roller skis (Pro-Ski C2, Sterners, Nyhammar, Sweden). All of the participants were well accustomed to treadmill roller skiing, both as part of their regular training and from their involvement in previous testing. They visited the laboratory on two occasions: a) first, to carry out a preliminary test to determine VO_2max_, maximal heart rate (HR_max_) and the linearity of the velocity-VO_2_ relationship for DIA and DP; and b) two days later to perform a continuous experimental protocol involving DIA and DP at race intensity.

### The preliminary test

The velocity required to obtain 90% VO_2max_ with DIA and 90% peak oxygen uptake (VO_2peak_) with DP were established employing separate linear graded protocols. In the case of DIA, VO_2_ was determined at several submaximal workloads separated by a one-minute break at a fixed incline of 6.5°, with an initial velocity set to 6 km∙h^−1^. The increase for the submaximal workloads was 2 km∙h^−1^ using four minute long bouts. During this submaximal protocol capillary blood samples were taken between workloads for determination of lactate and the onset of blood lactate accumulation (OBLA) (4.0 mmol · l^−1^) (Ivy et al. 1980; Sjödin and Jacobs 1981). VO_2max_ values were established using a ramp protocol starting at 11 km∙h^−1^ at an incline of 4°, with an increase in gradient by 1° every minute. One day later the VO_2peak_ for DP was obtained starting at a fixed inclination of 1°, initial velocity of 17 km · h^−1^, and a 2 km · h^−1^ increase in workload every fourth minute, until exhaustion. Using the means of the three highest consecutive VO_2_ values during the last minute at each submaximal workload, with a total sampling time of 30 s (mixing chamber), simple linear regression was applied to calculate the velocities required to achieve the targets of 90% VO_2max_ (DIA_LIN_) and 90% VO_2peak_ (DP_LIN_).

### Experimental procedures

The subjects reported to the laboratory two hours before starting the experimental protocol and were fitted with the EMG system and, thereafter, to normalize the amplitudes of the signals obtained, performed maximal voluntary contractions (MVC) with each muscle. Next, they rested in a supine position while the three catheters were inserted under local anesthesia (Carbocain 1%), utilizing an ultrasound system (Mindray DigiPrince DP-6600, Mindray Bio-Medical Electronics Co., Shenzhen, China) to visualize the vessels, as reported elsewhere (Calbet et al. 2005). For sampling arterial blood, a 16-gauge catheter (Arrow ES-04401, 300 mm, Arrow International Inc., Reading, PA) was inserted percutaneously using the Seldinger technique. Thereafter, a 20-gauge catheter (Arrow ES-04150, 120 mm) was inserted into the right femoral vein for collection of venous blood. Finally, another 16-gauge catheter (Arrow ES-04401, 300 mm) was inserted into an antecubital vein and then advanced into the subclavian vein for additional sampling of venous blood. (For further details, see Björklund et al. (2010)). All three catheters were sutured to the skin to minimize the risk of movement during exercise and connected to tubing (Alaris G420-B, 2000 mm, Cardinal Health, Switzerland) with a three-way stopcock and filled with isotonic saline containing heparin (100 E∙mL^−1^).

For the actual testing, inclines of 6.5° and 1° and velocities calculated to elicit 90% VO_2max_ and VO_2peak_ were used for DIA (11.2 ± 0.3 km∙h^−1^) and DP (24.3 ± 1.1 km∙h^−1^), respectively. The continuous experimental protocol was preceded by a 20 min warm-up with DIA and DP at 60 – 90% VO_2max_/VO_2peak._ The test itself consisted of 3 min of DIA at 90% of VO_2max_, followed by 3 min of DP at 90% VO_2peak_ and, finally, a second 3-min bout of DIA at 90% VO_2max_.

### Physiological measurements

Respiratory variables were monitored with the mixed expired procedure, employing an ergo-spirometry system (AMIS 2001 model C, Innovision A/S, Odense, Denmark) equipped with pneumotachograph to measure inspiratory flow. This system was calibrated with the standardized procedure described by Björklund et al. (2007, 2010). VO_2_, VCO_2_ and the inspired minute ventilation (V_E_) were monitored continuously and the average VO_2_ during the final 30 s at each workload calculated.

Heart rate (HR) was followed continuously using the Polar S610 monitor (Polar Electro Oy, Kempele, Finland) in combination with the metabolic cart. During the final minute of exercise at each bout, arterial and venous blood samples (1.5 ml) were collected anaerobically into syringes (PICO 50, Radiometer, Copenhagen, Denmark) containing heparin and then analyzed immediately for hemoglobin concentration [Hb], pH, PO_2_, PCO_2_, oxygen saturation (SO_2_) (ABL 80; Radiometer, Copenhagen, Denmark) and lactate (Biosen 5140, EKF-diagnostic GmbH, Magdeburg, Germany). The O_2_ content of arterial (CaO_2_) and venous (femoral: CfvO_2_; subclavian: CsvO_2_) blood was calculated from the SO_2_ and [Hb] as follows: (1.34 × [Hb] × SO_2_) + (0.003 × PO_2_) (Calbet et al. 2005).

### Force measurements

Each participant used custom-made carbon-fiber racing poles adjusted to his preferred length. The ground reaction force was measured by a strain gauge force transducer mounted directly below the grips of these poles (Hottinger–Baldwin Messtechnik GmbH, Darmstadt, Germany). Plantar ski reaction forces were recorded at 100 Hz by a Pedar mobile system (Novel GmbH, Munich, Germany). These systems for determination of the pole and plantar forces were validated and calibrated utilizing procedures described previously (Holmberg et al. 2005).

### EMG measurements

The EMG activity at the surface of the triceps brachii, latissimus dorsi, rectus abdominis, gluteus maximus, and gastrocnemius (medial head) muscles on the right side of the body were recorded employing pre-gelled bipolar Ag/AgCl surface electrodes (Skintact, Leonhard Lang GmbH, Innsbruck, Austria). Prior to fixation of these electrodes, the skin was shaved, abraded lightly, degreased, and disinfected with alcohol. The electrodes were positioned in parallel on the surface of the muscle belly in the direction of the fibers and 30 mm apart, in accordance with international standards (Hermens et al. 1999). The reference electrode was attached to the tibia. The active and reference electrodes for each muscle were connected to a single differential amplifier (base gain 500; input impedance >100 MΩ; common mode rejection ratio >100 dB; input range ±10 mV).

### Processing the EMG signals

Prior to the calculation of EMG parameters, the raw signals were band-pass filtered digitally (10–400 Hz; Butterworth 2^nd^ order) to remove both low- and high-frequency noise (Winter 1990). After full wave rectification of the signals the integrated and root mean square electromyography (IEMG and EMG_RMS_ respectively) values for defined phases of exercise were calculated for all of these muscles. After training several times, MVC was performed with each muscle (Acierno et al. 1995; Holmberg et al. 2005). The recording and processing of MVC data were carried out according to Björklund et al. (2010). Statistical analysis (see further below) for IEMG and EMG_RMS_ was performed using the sum, respectively the mean values for the arm (triceps brachii, and latissimus dorsi) and leg muscles (gastrocnemius, gluteus maximus, rectus femoris).

### Collection and analysis of biomechanical data

EMG signals and pole forces were amplified with a telemetric recording system (TeleMyo 2400T G2, Noraxon, Scottsdale, AZ) and simultaneously stored (at a sampling rate of 3000 Hz) on a computer via an A/D converter card. Synchronization of the measurement of EMG signals and pole and plantar forces was achieved with a signal produced by the Pedar mobile system. The mean values for the ten successive cycles during the final 30 s of exercise at the three different bouts were subjected to statistical analysis.

Within each cycle of the DIA skiing, the following four phases were analyzed: 1–2) poling and recovery of the arms; 3) leg-gliding (starting from placement of the roller ski and ending when the leg force was minimal immediately prior to the increase in force associated with the push-off); and 4) the push-off and recovery of the legs. One such cycle was defined as extending from a pole plant to the next pole plant on the same side of the body. A cycle of DP skiing was divided into the poling and recovery phases. The average pole and plantar forces were calculated by dividing the integrated signal by the cycle time. All data were processed using the IKE-master software (IKE-Software Solutions, Salzburg, Austria).

### Statistical analyses

All sets of data exhibited a Gaussian distribution, as confirmed by applying the Shapiro-Wilk test. Values are presented as means ± SD. A two way repeated-measures ANOVA (skiing technique × sampling site) was employed to test for differences between skiing techniques and sampling sites, as well as between biomechanical parameters associated with the upper- and lower-body. When a significant interaction was observed, paired sampled t-tests or a one-way ANOVA with repeated measures was performed. When only a single measurement at each workload was performed, a one-way ANOVA with repeated measures was used. A Bonferroni Post-Hoc test was performed on global effects identified by ANOVA. Pair-wise relationships between variables were compared using Pearson’s product moment correlation coefficient. A level of α < 0.05 was considered to be statistically significant in all cases. All statistical analyses were performed with the SPSS 18.0 program (IBM Corporation, Somers, NY).

## Results

### General observations

**Figure 1:**
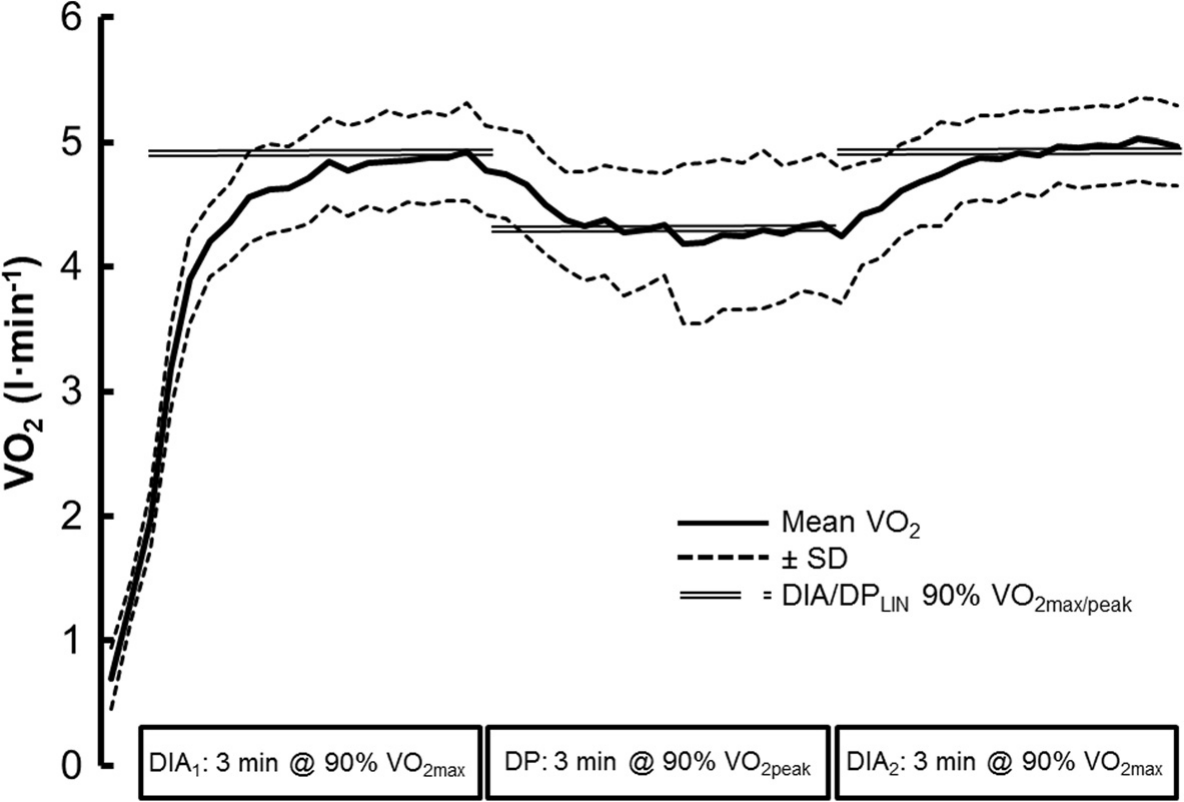
**VO**_**2**_ **values (means ± SD) during the three sessions of roller skiing on a treadmill.** The mean calculated 90% VO_2max_ (DIA_LIN_) and 90% VO_2peak_ (DP_LIN_) are illustrated by the double line.

The calculated VO_2_ for DIA and the averaged measured VO_2_ during the first and second bout of DIA skiing and were 4.84 ± 0.95, 4.89 ± 0.13 and VO_2_ 4.99 ± 0.11 l · min^−1^, respectively (*P* = 0.081) (Figure 1). Similarly, the calculated VO_2_ for DP and the averaged measured VO_2_ during DP the skiing did not differ (4.35 ± 0.36 and 4.36 ± 0.49 l · min^−1^, respectively; *P* = 0.94). The cardio-respiratory and biomechanical values are documented in Table 2.

**Table 2:** **Cardio-respiratory and biomechanical values for our 8 well-trained male cross-country skiers while roller-skiing with the diagonal stride (DIA) or double poling (DP) technique at 90% VO**_**2max/peak**_ **on a treadmill**

	**First session of DIA**	**DP**	**Second session of DIA**	***F*** **-value**	***P*** **-value**	**Power**
Cardio-respiratory parameters						
VO_2_ (l⋅min^−1^)	4.89 ± 0.36^#^	4.36 ± 0.49^*†^	4.99 ± 0.11^#^	*F*_(1.2,8.6)_ = 35.3	*P* < 0.001	1.000^$^
Heart rate (beats · min^−1^)	174 ± 4^†^	168 ± 7^†^	181 ± 4^*#^	*F*_(2,14)_ = 14.7	*P* < 0.001	0.994
V_*E*_ (l · min^−1^)	152 ± 8^†^	155 ± 18	172 ± 14^*^	*F*_(2,14)_ = 8.52	*P* = 0.004	0.921
O_2_ pulse (ml per beat)	28.1 ± 2.4^#^	25.8 ± 3.1^*^	27.3 ± 2.2	*F*_(1.1,7.8)_ = 5.77	*P* = 0.041	0.581^$^
Breaths · min^−1^	47 ± 2^#†^	52 ± 3^*^	53 ± 7^*^	*F*_(1.2,8.3)_ = 6.79	*P* = 0.027	0.672^$^
V_*T*_ (ml)	3.24 ± 0.23^#^	2.97 ± 0.39^*†^	3.23 ± 0.27^#^	*F*_(2,14)_ = 5.43	*P* = 0.018	0.760
Respiratory exchange ratio	1.07 ± 0.06	1.11 ± 0.06	1.09 ± 0.04	*F*_(2,14)_ = 1.74	*n.s.*	
V_*E*_/VO_2_	31.2 ± 1.9^#†^	35.6 ± 2.1^*^	34.9 ± 3.8^*^	*F*_(2,14)_ = 14.7	*P* < 0.001	0.994
V_*E*_/VCO_2_	29.1 ± 1.1^#†^	31.9 ± 1.4^*^	32.0 ± 2.7^*^	*F*_(2,14)_ = 10.7	*P* = 0.002	0.966
Biomechanical parameters						
Speed (km∙h^−1^)	11.2 ± 0.3	24.3 ± 1.1	11.2 ± 0.3			
Cycle rate (Hz)	0.79 ± 0.02^#^	0.87 ± 0.02^*†^	0.78 ± 0.01^#^	*F*_(2,14)_ = 13.1	*P* = 0.001	0.985
Cycle length (m)	3.94 ± 0.06^#^	7.82 ± 0.23^*†^	4.00 ± 0.05^#^	*F*_(2,14)_ = 211	*P* = 0.001	1.000
Cycle time (s)	1.28 ± 0.07^#^	1.16 ± 0.00^*†^	1.30 ± 0.06^#^	*F*_(2,14)_ = 12.3	*P* = 0.001	0.979
Recovery time for the arms (s)	0.75 ± 0.06^#^	0.80 ± 0.06^*†^	0.75 ± 0.05^#^	*F*_(2,14)_ = 4.91	*P* = 0.028	0.693
Relative arm recovery time (% cycle time)	58.7 ± 2.5^#^	68.9 ± 1.6^*†^	57.9 ± 2.7^#^	*F*_(2,14)_ = 155	*P* = 0.001	1.000
Peak pole force (N)	101 ± 7.4^#^	287 ± 45.2^*†^	96.0 ± 5.5^#^	*F*_(2,14)_ = 22.3	*P* = 0.001	1.000
Recovery time for the legs (s)	0.50 ± 0.04		0.52 ± 0.03		*n.s.*	
Peak leg force (N)	666 ± 87		673 ± 80		*n.s.*	
Impulse of pole force (Ns)	33.6 ± 5.9	39.4 ± 9.6	30.9 ± 3.7	*F*_(2,14)_ = 4.49	*P* = 0.035	0.651
Impulse of foot force (Ns)	481 ± 100	392 ± 54^†^	482 ± 79^#^	*F*_(2,14)_ = 9.47	*P* = 0.003	0.937

The values are presented as means ± SD. VO_2_, oxygen uptake; V_E_, minute ventilation; V_*T*_, tidal volume; Bonferroni correction was used to check for differences between time-points. A One-Way repeated ANOVA was used to compare the responses and a paired Student’s t-test was when only two time-points were compared.

*Statistically different from DIA_1._

^#^Statistically different from DP.

^†^Statistically different from DIA_2_.

^$^Greenhouse-Geisser-adjusted ANOVA, since the sphericity was violated.

*n.s.* not statistically significant.

### Muscle activation

**Figure 2:**
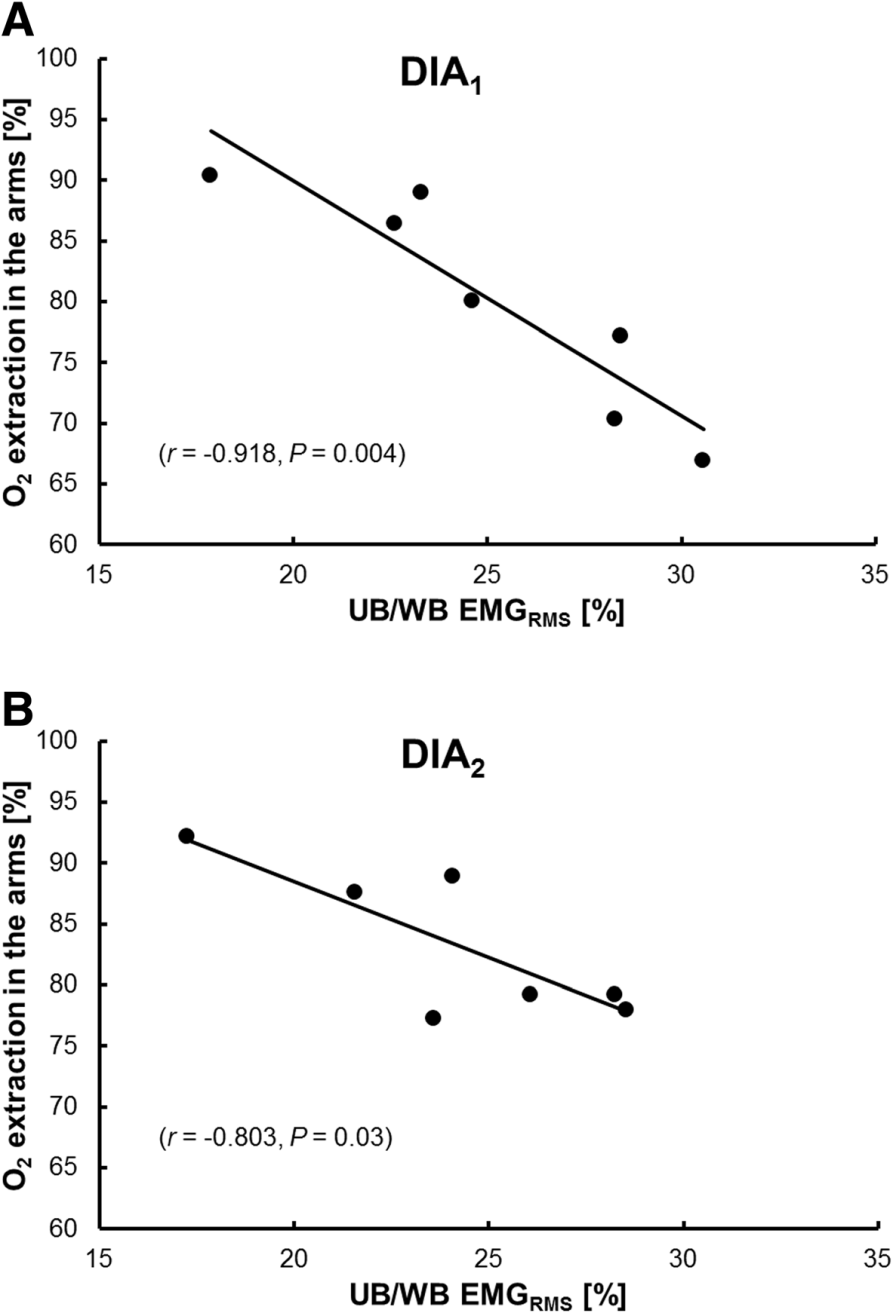
**The relationship between extraction of O**_**2**_ **in the arms and the ratio of upper- (UB) and whole-body EMG**_**RMS**_ **during the first (A) (DIA**_**1**_ **) and second sessions (B) (DIA**_**2**_ **) of diagonal stride skiing.**

During the poling phase the EMG_RMS_ for the arms was 36.7 and 49.7% higher with DP than during the first and second sessions of DIA skiing, respectively (both *P* < 0.05). Especially during DP high EMG_RMS_ values of 99.7 ± 12.9 %MVC (range: 69–159 %MVC) were found. The relative EMG_RMS_ for the arms (arms/whole body) for the entire cycle was 32.7% lower during DP (*P* < 0.001) than the first session of DIA (Table 3). The sums of the IEMG and average EMG (AEMG) for all of the muscles analyzed during both sessions of DIA were similar and greater than DP (IEMG: DIA_1_: 107 ± 24; DP: 86 ± 26; DIA_2_: 106 ± 25 %MVCs; AEMG: 93 ± 27, DP 72 ± 20, DIA2 79 ± 22 %MVC/s; *P* < 0.01 in both cases). The ratio of upper-/whole-body EMG_RMS_ was inversely correlated with arm O_2_ extraction in the arms during both sessions of DIA (*r* = −0.918, *P* = 0.004 and *r* = −0.803, *P* = 0.03 respectively) (Figure 2A-B), whereas there was no such correlation in the case of DP.

**Table 3:** **The activities of arm and leg muscles during the entire cycle and the poling and recovery phases of the first and second sessions of DIA and the DP skiing at 90% of VO**_**2max/peak**_

	**First session of DIA**	**DP**	**Second session of DIA**	***F*** **-value**	***P*** **-value**	**Power**
IEMG for the entire cycle arms (%MVCs)	29.1 ± 9.2	25.7 ± 11.1	27.7 ± 8.0	^a^ *F*_(2,14)_ = 9.29	*P* = 0.004	0.932
IEMG for the entire cycle legs (%MVCs)	16.1 ± 3.5^#^	11.6 ± 3.8*^†^	16.0 ± 3.2^#^	^b^ *F*_(1,7)_ = 15.64	*P* = 0.007	0.906
				^c^ *F*_(2,14)_ = 0.25	*n.s*	
EMG_RMS_ poling phase, arms (%MVC)	72.9 ± 8.9^#^	99.7 ± 12.9*^†^	66.6 ± 10.7^#^	^a^ *F*_(1,7)_ = 6.66	*P* = 0.042	0.580
EMG_RMS_ push-off phase legs (%MVC)	44.6 ± 3.5		43.0 ± 3.6	^b^ *F*_(1,7)_ = 7.44	*P* = 0.034	0.626
				^c^ *F*_(1.7)_ = 1.13	*n.s.*	
EMG_RMS_ recovery phase, arms (%MVC)	14.2 ± 2.4	14.5 ± 3.1	15.1 ± 2.2	^a^ *F*_(2,14)_ = 39.5	*P* = 0.001	1.000
EMG_RMS_ recovery phase, legs (%MVC)	31.4 ± 2.3^#^	18.0 ± 2.8*^†^	30.5 ± 2.0^#^	^b^ *F*_(2,14)_ = 51.1	*P* = 0.001	1.000
				^c^ *F*_(4,28)_ = 5.24	*P* = 0.023	0.723
EMG_RMS_ ratio, entire cycle, arms/whole body (%)	25.1 ± 1.6^#^	16.9 ± 2.4*^†^	24.2 ± 1.5^#^	*F*_(1,7)_ = 13.0	*P* = 0.001	0.984

The values presented are means ± SD. IEMG, integrated EMG; EMG_RMS_, EMG root mean squared; MVC, maximal voluntary contraction. A Two-Way repeated ANOVA (3 × 2) was used to compare the responses during the two sessions of DIA and DP. Since there was no push-off phase for the legs during DP, a 2 × 2 Two-Way repeated ANOVA was used to compare the EMG_RMS_ for the poling and push-off phases. A One-Way repeated ANOVA was used in the case of the EMG_RMS_ ratio and Bonferroni correction was used to compare different time-points, with paired Student’s t-test instead when only two time-points were compared.

*Significantly different from DIA_1._

^#^Significantly different from DP.

^†^Significantly different from DIA_2_.

^a^Main effect: exercise intensity.

^b^Main effect: extremity.

^c^Interactive effect: exercise intensity x extremity.

*n.s.* not statistically significant.

### Force and kinematics

Cycle rate and peak pole force were higher during DP than during both sessions of DIA (both *P* < 0.001), during which these values were similar. The impulse of pole forces was similar in all three sessions (*P* > 0.05). The absolute and relative (% cycle time) recovery times for the arms were longer with DP, with no difference between the two sessions of DIA (Table 2) (*P* > 0.05). During DIA, O_2_ extraction in the legs was negatively correlated to minimal leg force during ground contact (the end of the gliding phase) (*r* = −0.821, *P* = 0.024) and to the IEMG and EMG_RMS_ for the entire cycle in the lower-body (*r* = −0.782, *P* = 0.038 and *r* = −0.794, *P* = 0.033, respectively). During DP, O_2_ extraction in the legs was negatively correlated to minimal leg force (*r* = −0.941, *P* = 0.002) and positively associated with cycle length (*r* = 0.862, *P* = 0.013). O_2_ extraction in the arms during DP was correlated to the cycle characteristics length (*r* = 0.941, *P* = 0.005), rate (*r* = −0.853, *P* = 0.031) and time (*r* = 0.865, *P* = 0.026), as well as absolute recovery time during the poling phase (*r* = 0.850, *P* = 0.032).

### Overall extraction of O_2_

**Figure 3:**
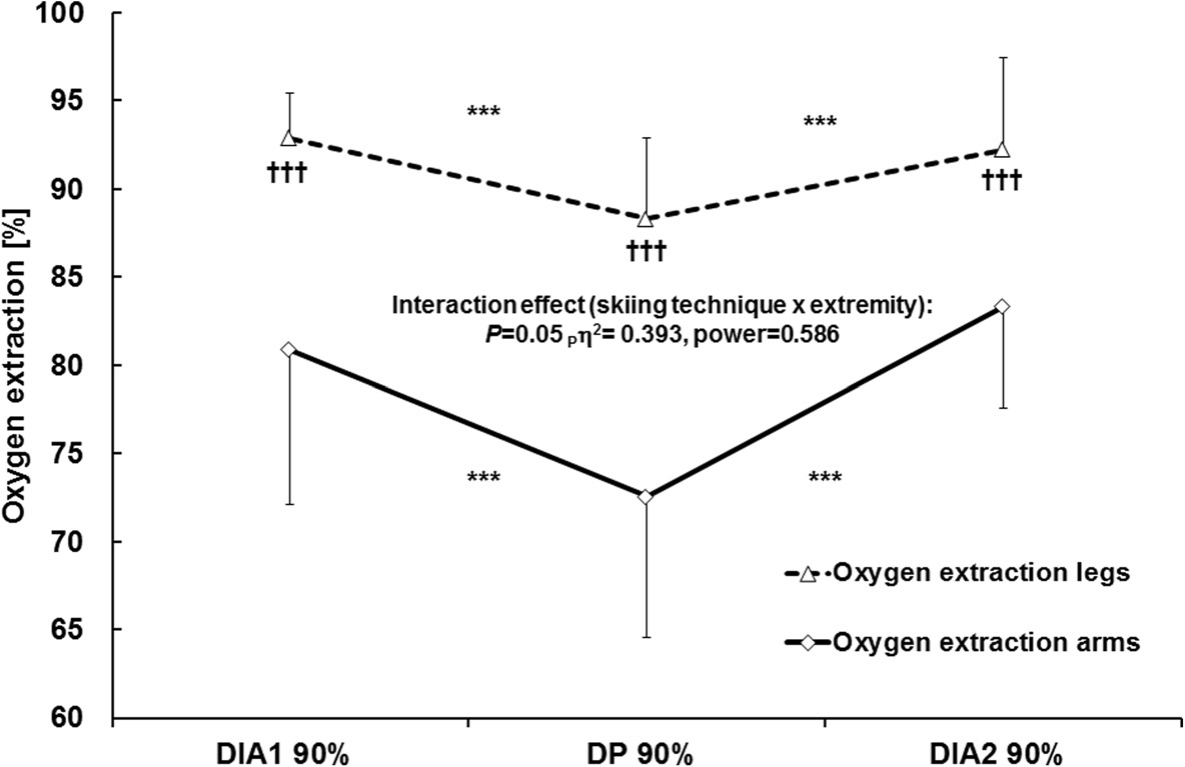
**O**_**2**_ **extraction in the arms and legs during the first and second sessions of diagonal stride skiing and the intervening double poling at 90% VO**_**2max/peak**_ **.** The values presented are as means ± SD. The *P*-values, effect size (_*p*_η^2^), and observed power obtained with the two-way ANOVA (skiing technique × extremity) are presented. ***P < 0.001 in comparison to DP and ††† *P* < 0.001 in comparison to the arms.

O_2_ extraction was higher in the legs than the arms at all workloads (*F*_1,7_ = 25.9, *P* = 0.002) and was lower in all limbs during DP than the two session of DIA (*F*_2,14_ = 10.3, *P* = 0.002 and F_2, 14_ = 10.4, *P* = 0.002, respectively), with no difference between the latter (leg *P* = 1.00 and arm *P* = 0.21). There was an interaction between skiing technique and sampling site (*F*_2,14_ = 3.89, *P* = 0.05), which might reflect the more pronounced reduction in O_2_ extraction in the arms during DP than both sessions of DIA (*F*_2,14_ = 13.31, *P* = 0.001) (Figure 3).

### Blood levels of lactate and pH

**Figure 4:**
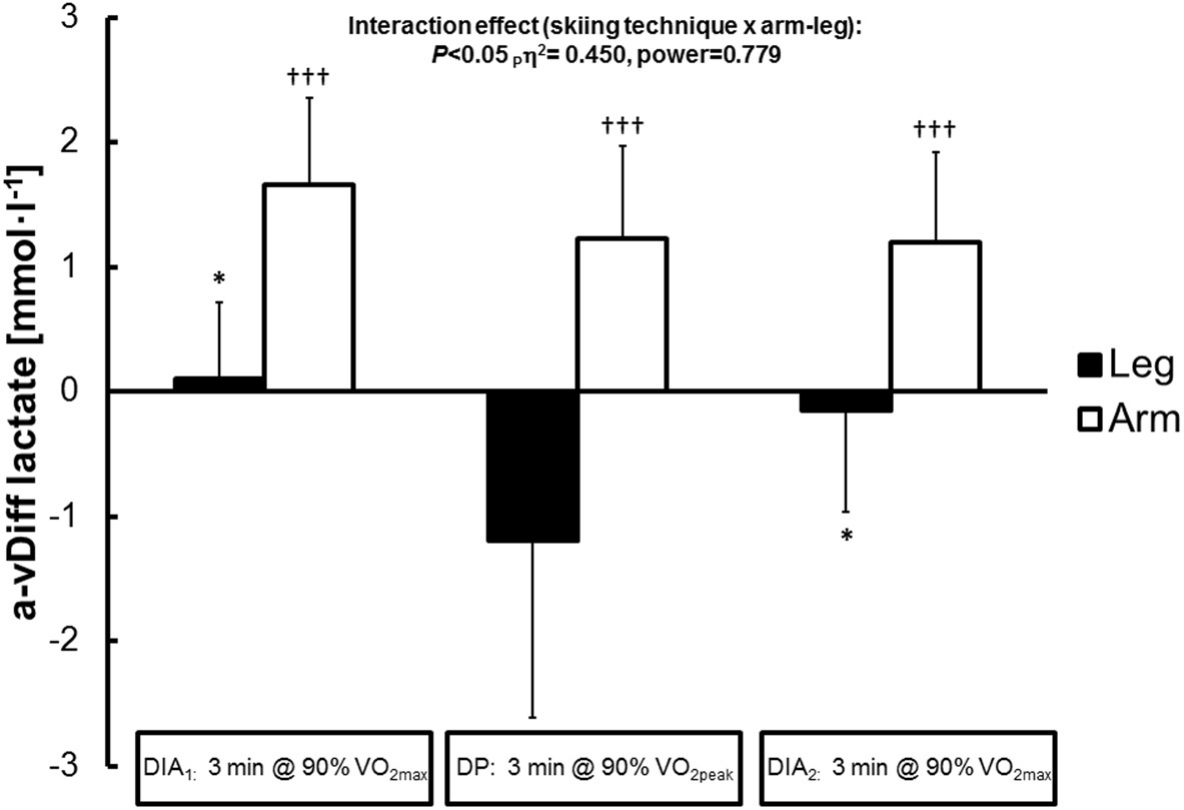
**The arterial-venous difference in blood levels of lactate in the legs and arms during the first and second sessions of diagonal stride skiing and the intervening double poling at 90% VO**_**2max/peak**_ **.** The values presented are means ± SD. The *P*-values, effect size (_*p*_η^2^), and observed power obtained with the two-way ANOVA (skiing technique × extremity) are presented. *P < 0.05 in comparison to DP and ††† *P* < 0.001 in comparison to the legs.

The arterial-venous difference in blood level of lactate in the legs was lowest (actually negative) during DP and similar during both sessions of DIA (*F*_2,14_ = 5.92, *P* = 0.014). In contrast, in the case of the arms this difference remained constant (*F*_2,14_ = 2.17, *P* = 0.163) (Figure 4). The absolute blood level of lactate rose from the first session of DIA to DP and again to the second session of DIA, being significantly higher in the subclavian vein than in either the femoral artery or vein throughout the entire test (*P* < 0.05).

The blood level of bicarbonate fell to an equal extent at all three sampling sites for each successive workload (*P* < 0.05). The pH was lower in both the subclavian and femoral veins than the femoral artery during all three sessions, with the lowest values for all sampling sites occurring during the second session of DIA (*P* < 0.01). The PCO_2_ remained constant throughout the test and was higher in both the femoral and subclavian vein than the femoral artery (*P* < 0.05) (Table 4).

**Table 4:** **Lactate, pH and gases in the blood during roller-skiing with the diagonal stride (DIA) or double-poling (DP) technique at 90% VO**_**2max/peak**_

**Variable**	**Resting**	**First session of DIA**	**DP**	**Second session of DIA**	***F*** **-values,** ***P*** **-values, observed power**
**[Lactate], mmol · l** ^**−1**^					
Femoral artery	0.8 (±0.1)	4.6 (±0.9)^¤†^	7.0 (±1.6)^*†^	7.9 (±2.0)^*¤†^	^a^ *F*_(2,14)_ = 46.4, *P* < 0.001, power = 1.0
Femoral vein	0.7 (±0.2)	4.7 (±0.8)^¤†^	5.8 (±1.7)^†^	7.8 (±1.8)^*¤†^	^b^ *F*_(2,14)_ = 29.4, *P* < 0.001, power = 1.0
Subclavian vein	0.8 (±0.3)	6.3. (±1.4)^¤$‡^	8.2 (±1.6)^*$‡^	9.1 (±2.0)^*$‡^	^c^ *F*_(4,28)_ = 5.17, *P* < 0.01, power = 0.94
**[Bicarbonate], mmol · l** ^**−1**^					
Femoral artery	23.6 (±1.9)	19.2 (±1.0)^¤^	17.8 (±1.4)^*^	16.9 (±1.3)^*^	^a^ *F*_(2,14)_ = 51.7, *P* < 0.001, power = 1.0
Femoral vein	23.5 (±1.6)	19.4 (±1.3)^¤^	17.8 (±1.6)^*^	16.6 (±1.2)^*¤^	^b^ *F*_(2,14)_ = 0.081, *n.s.*
Subclavian vein	23.7 (±1.3)	19.4 (±1.1)^¤^	17.4 (±0.8)	16.5 (±1.2)^*¤^	^c^ *F*_(4,28)_ = 0.212, *n.s.*
**pH**					
Femoral artery	7.42 (±0.01)	7.37 (±0.02)^¤‡†^	7.34 (±0.03)^*†‡^	7.31 (±0.04)^*¤†‡^	^a^ *F*_(2,14)_ = 32.97, *P* < 0.001, power = 1.0
Femoral vein	7.38 (±0.02)	7.27 (±0.02)^¤$†^	7.23 (±0.04)^*$^	7.19 (±0.03)^*¤$^	^b^ *F*_(2,14)_ = 384, *P* < 0.001, power = 1.0
Subclavian vein	7.37 (±0.03)	7.24 (±0.03)^¤$‡^	7.21 (±0.03)^*$^	7.19 (±0.03)^*¤$^	^c^ *F*_(4,28)_ = 5.68, *P* < 0.01, power = 0.95
**O**_**2**_ **content, ml · l** ^**−1**^					
Femoral artery	190 (±15.2)	193 (±15.3)^†‡^	192 (±27.8)^†‡^	200 (±11.0)^†‡^	^a^ *F*_(2,14)_ = 1.51, *n.s.*
Femoral vein	100 (±23.9)	13.4 (±4.6)^$†^	22.4 (±9.9)^$†^	15.6 (±10.7)^$†^	^b^ *F*_(2,14)_ = 428, *P* < 0.001, power = 1.0
Subclavian vein	120 (±29.8)	36.1 (±15.0)^$‡^	51.0 (±12.6)^$‡^	33.2 (±11.0)^$‡^	^c^ *F*_(4,28)_ = 2.52, *n.s.*
**SO**_**2**_ **(%)**					
Femoral artery	98.3 (±0.8)	94.8 (±2.0)^¤†‡^	96.0 (±1.7)^*†‡^	94.3 (±2.0)^¤†‡^	^a^ *F*_(2,14)_ = 16.28, *P* < 0.001, power = 0.99
Femoral vein	51.1 (±11.1)	6.5 (±2.4)^¤$†^	11.2 (±5.2)^*$†^	7.5 (±5.2)^¤$†^	^b^ *F*_(2,14)_ = 1294, *P* < 0.001, power = 1.0
Subclavian vein	57.0 (±17.9)	16.9 (±7.5)^$‡^	22.7 (±6.1)^$‡^	14.7 (±5.3)^¤ $‡^	^c^ *F*_(4,28)_ = 4.28, *P* < 0.01, power = 0.87
**PO**_**2**_ **(mm Hg)**					
Femoral artery	111 (±15.2)	77.3 (±10.8)^¤†‡^	88.1 (±11.4)^*†‡^	78.9 (±9.8)^¤†‡^	^a^ *F*_(2,14)_ = 15.47, *P* < 0.001, power = 0.99
Femoral vein	27.5 (±4.8)	8.8 (±2.3)^¤$†^	12.6 (±3.6)^*$†^	9.8 (±4.5)^¤$†^	^b^ *F*_(2,14)_ = 471, *P* < 0.001, power = 1.0
Subclavian vein	32.1 (±8.7)	16.3 (±4.2)^$‡^	20.0 (±3.2)^$‡^	15.8 (±3.7)^$‡^	^c^ *F*_(4,28)_ = 4.21, *P* < 0.05, power = 0.86
**PCO**_**2**_ **(mm Hg)**					
Femoral artery	34.9 (±4.0)	30.5 (±2.6)^†‡^	29.8 (±2.3)^†‡^	29.3 (±2.3)^†‡^	^a^ *F*_(2,14)_ = 2.04, *n.s.*
Femoral vein	43.5 (±4.2)	55.1 (±4.1)^$^	54.8 (±3.2)^$^	59.0 (±4.9)^$^	^b^ *F*_(2,14)_ = 179, *P* < 0.001, power = 1.0
Subclavian vein	45.5 (±5.3)	60.3 (±7.0)^$^	57.9 (±5.1)^$^	59.8 (±2.9)^$^	^c^ *F*_(4,28)_ = 2.26, *n.s.*

The values shown are means (±SD). PO_2_, partial pressure of oxygen; PCO_2_, partial pressure of carbon dioxide; SO_2_, oxygen saturation.

The *F-* and *P*-values are for two-way ANOVA (3 × 3) analysis of exercise intensity (90% VO_2max/peak_) x sampling site.

^a^Main effect between intensities. ^$^Significantly different to femoral artery. ^*^Significantly different to DIA_1_90%.

^b^Main effect between sampling sites. ^‡^Significantly different to femoral vein. ^¤^Significantly different to DP90%.

^c^Intensity by sampling site interactive effect. ^†^Significantly different to subclavian vein.

*n.s.* not significant.

### The breathing pattern

During DP the cycle and breathing rates were tightly coupled (i.e. entrainment), with 52 breaths · min^−1^ (0.87 Hz) being similar to the cycle rate (a ratio of 1:1) (*r* = 0.89, *P* = 0.007). In the case of DIA, the rate of breathing was similar to the cycle rate during the first session (approximately 0.79 Hz), but greater than cycle rate during the second (0.88 Hz vs. 0.78 Hz, *P* = 0.022). Furthermore, there was no correlation between the breathing and cycle rates during either session of DIA (*r* = 0.23 and *r* = 0.18 both *P* > 0.05).

## Discussion

The major findings of the study using repeated high-intensity DIA bouts interspersed with high-intensity DP were: 1) DP did not alter the redistribution in muscle activation between arms and legs and any further biomechanical variables between DIA_1_ and DIA_2_. 2) O_2_ extraction in both arms and legs was unaffected by DP as O_2_ extraction was unchanged between DIA_1_ and DIA_2_. However, arm O_2_ extraction decreased during DP compared to DIA despite higher muscle activation, a three-fold increase in pole ground reaction force and an augmented blood lactate concentration in the arms. 3) Leg O_2_ extraction was related to a greater unloading of the lower body by a more dynamic lower body work during DP and both DIA, while arm O_2_ extraction was associated with cycle characteristics (e.g. cycle length, cycle rate, swing time) for DP only. Interestingly, a greater ratio of upper- to whole-body muscle activation at both DIA_1_ and DIA_2_ was associated to an attenuated arm O_2_ extraction. 4) Pulmonary VO_2_ did not increase over time as VO_2_ for DIA_1_ and DIA_2_ was similar. However, there was an apparent acid base disturbance from DIA_1_ to DIA_2_ reflected by a decrease in pH, bicarbonate and an increase in lactate both systemically and for venous arm and leg blood.

### Effects of DP on upper to lower body muscle activation during DIA

Despite increased muscle activation and peak pole forces during DP compared to DIA (~ + 40%, +185%) no changes in regards of muscle activation distribution between upper- and lower-body and cycle characteristics for DIA_2_ compared to DIA_1_ were induced. Previous data from repeated sprint cross-country skiing on snow, using a higher exercise intensity than in the current study, demonstrated a decrease in upper body force and power output during DP (Zory et al. 2009). This decrease was accompanied by a decrease in performance. Our study was carried out on a treadmill using preset velocities where the skier was not able to adjust the velocity. However, this does not explain the absence in redistribution between upper and lower body as both arms and legs are used for propulsion during DIA. Also, it could be that the intensity was not strenuous enough for these well-trained skiers in accordance with a previous report that elite athletes withstand fatigue better to maintain neuromuscular control (Bonacci et al. 2011). Furthermore, the increased muscle activation during DP compared to DIA was specifically during the poling phase while for the entire movement cycle as well as the recovery phase no difference was observed. Therefore, even though the high demands on the upper body during high-intensity DP the upper-body contribution during DIA seems to be too small to necessitate a redistribution of upper to lower body work.

### O_2_ extraction DP vs. DIA

One of the novel findings was the decreased arm O_2_ extraction during DP compared with DIA, although increased upper-body muscle activation (up to ~100% of MVC in DP vs. 70% of MVC in DIA). The result of a lower arm O_2_ extraction during DP than DIA have previously been demonstrated by Calbet et al. (2005) using a lower exercise intensity (76 vs. 90% of VO_2max/peak_). Furthermore, there was an interaction effect caused by a more pronounced decrease in O_2_ extraction in arms than legs when switching from DIA_1_ to DP and back to DIA_2_. The reason for a lower arm O_2_ extraction during DP has been shown, at least in part, to be due to a possible mechanical hindrance caused by high pole force generation and high arm muscle activity (~96% MVC) that hinders further elevation of O_2_ extraction (Stöggl et al. 2013). The current study supports this finding, based on the reduction in arm O_2_ extraction despite increased pole forces and upper body muscle activation when comparing DP with DIA.

Another explanation for the attenuated arm O_2_ extraction during DP could be the decrease in total activated muscle mass, as reflected by the increased sum IEMG and AEMG values during DIA compared to DP. Mortensen et al. (2008) demonstrated that when activating a larger muscle mass (two-legged supra maximal cycling compared with one-legged exercise), locomotor skeletal muscle perfusion leveled off with a concomitant larger increase in O_2_ extraction. Furthermore, comparisons between arm cycling vs. combined arm and leg cycling shows a decrease in arm blood flow when adding leg to arm exercise but at the same time increased O_2_ extraction in the arms (Volianitis and Secher 2002). More specifically, in a study by Calbet et al. (2004) arm blood flow was substantially higher using DP than DIA although at a similar cardiac output. Therefore, in the current study high-intensity DIA likely induce additional central cardiovascular stress due to an increased activated total muscle mass than DP – as demonstrated by the augmented sum IEMG and AMEG values - and thus induces a higher O_2_ extraction in both arms and legs to match the increased O_2_ demand.

### Relationships between biomechanical variables and O_2_ extraction

Skiers who unloaded their legs to a greater extent (leg force minima) at the end of the gliding phase prior to leg push-off during DIA and prior to pole plant during DP extracted more O_2_ in the legs. The last finding is in accordance with a previous study from our group were leg force minima was related to both O_2_ extraction in the legs and arms at 90% and 70% of VO_2peak_ during DP (Stöggl et al. 2013). Furthermore, this result fits nicely with the previous demonstration that modern DP involves more dynamic use of the lower body: a higher body position prior to pole plant; greater extension of the knee, hip and ankle joints; and distinct knee and hip flexion during the poling phase (Holmberg et al. 2005). In addition Stöggl et al. (2010) demonstrated that faster skiers had greater center of mass oscillation in vertical direction during DP and DIA being coupled with a more active and dynamic skiing technique. The aspect about coupling between greater unloading of the legs prior to the push-off phase during DIA and leg O_2_ extraction is novel. Therefore, more technically skilled skiers with more active and dynamic technique achieve a better O_2_ extraction.

Arm O_2_ extraction was negatively associated to cycle rate and positively related to cycle length and arm recovery time during DP, being in line with our previous findings during DP (Stöggl et al. 2013). This association between the decreased arm O_2_ extraction and cycle rate during DP might be influenced by coupling to limb blood flow. Accordingly, an increased cycle rate might increase the centrifugal force that amplifies the arm blood flow as shown in another study using arm exercise at a similar cycle rate as in the current study (0.75 Hz) (Sheriff et al. 2009). There is further support that an increased contraction frequency (in our case cycle rate) induces a greater muscle blood flow (Ferguson et al. 2001; Sheriff 2003). Possibly the increased cycle rate could induce a higher arterial pressure that increase or maintains a high blood flow although usually such a high muscle activity shown in the current study restricts blood flow (70–100% of MVC). For the legs on the other hand no relationship was noticed between cycle rate and leg O_2_ extraction which is in accordance with previous findings using isolated leg exercise (Ferguson et al. 2001).

### Excess VO_2_, muscle activation and lactate

It is suggested that it is not possible to perform a constant work rate at higher intensities that refers to a specific percent of VO_2max_ (Whipp 1994). One of the mechanisms proposed for this VO_2_ slow component is the magnitude and the time course of the increase in blood lactate, i.e. above the lactate threshold (Burnley and Jones 2007). In contrast to that, in the current study no changes in VO_2_ were found between DIA_1_ and DIA_2_ even though blood lactate steadily increased throughout the protocol (arterial: 4.6 to 7.9 mmol∙l^−1^). Comparable results were found in the study of Björklund et al. (2011) also demonstrating no increase in VO_2_ in world class cross-country skiers, when utilizing a variable intensity protocol containing 3 minutes exercise intensity of 90% of VO_2max_ interspersed with 6 minutes of 70% of VO_2max_ with a total duration of 24 min. One reason for the absence of a raise in VO_2_ from DIA_1_ to DIA_2_ in the current study might be due to the large amount of activated muscle mass and also already high O_2_ extraction (~90%) with DIA and DP making the potential for a further increase in VO_2_ diminutive.

### Respiration locomotion coordination

While the exercise intensity was similar between the two DIA bouts there was a shift in breathing pattern. First, V_*E*_ increased for the second DIA due to an increase in B_*f*_ (47 vs. 53 breaths · min^−1^) while no change in tidal volume. This is in accordance with previously shown data using DIA although at a lower exercise intensity (~76% VO_2max_) (Holmberg and Calbet 2007). Second, during the first DIA the B_*f*_ was similar as cycle rate (approximately 0.79 Hz), but at the second DIA workload there was a tachypneic shift, due to the elevated V_*E*_ caused by an increased B_*f*_ with a mean ratio of 1.13:1, (range 1.:1 – 1.4:1) (B_*f*_ 0.88 Hz vs. cycle rate 0.78 Hz). The cause for this asynchronic B_*f*_ to cycle rate ratio during DIA_2_ could be explained by the increased metabolic acidosis as the arterial blood lactate concentration went from 4.6 to 7.9 mmol · l^−1^ and the pH dropped from 7.37 to 7.31 between DIA_1_ to DIA_2_. However the skiers P_a_CO_2_ remained fairly stable and did not show signs of hypercapnia, nor was there a relation between the magnitude of asynchronic breathing and P_a_CO_2_. Further, even though B*f* increased between DIA_1_ and DIA_2_ the B*f* remained unchanged between DP and DIA_2_ although the latter of the two was performed with a lower cycle rate. The coordination between respiration – locomotion was therefore not fixed for DIA. However the breathing pattern in the current study confirms that during the single bout of DP there was a tight coupling between cycle rate and B_*f*_, i.e. entrainment, with a B_*f*_ of 52 breaths · min^−1^ equaling 0.87 Hz being similar to the cycle rate in DP (1:1). This 1:1 coupling could be exercise intensity dependent as shown previously during DP using a fixed cycle rate of 60 Hz were B_*f*_ and cycle rate was uncoordinated for lower speeds (12–18 km · h^−1^) while attaining a 1:1 ratio during 24 km · h^−1^ (Lindinger and Holmberg 2011).

### Limitations

Due to the lack of blood flow measurements it cannot be definitively concluded whether the differences in O_2_ extraction between the arms and legs and between DP and DIA are due to differences in blood flow and/or metabolic demands. Further arterial blood pressure measurements could add information regarding the effect of arm swing and cycle rate influence of both O_2_ extraction and blood flow. This link was especially pronounced during mainly upper-body work, i.e. DP in the current study.

## Conclusions

In well trained skiers, DP does not influence DIA VO_2_, at a simulated race intensity of ~90% of VO_2max/peak_. Furthermore, although high-intensity DP induce substantially higher arm muscle activation concurrent with a three-fold increase in pole force compared to DIA, it does not affect the redistribution of pole and foot forces, cycle characteristics and cardiorespiratory characteristics during the following DIA bout. This suggests that well trained skiers withstand the upper-body strain that high-intensity DP implies. The absence of an increase in VO_2_ from DIA_1_ to DIA_2_ despite a distinct increase of blood lactate questions the relation between blood lactate and excess VO_2_, and also strengthens the idea that VO_2_ slow component might not occur in well-trained athletes when using exercise modes where both legs and arms are at their upper limit of O_2_ extraction. Furthermore, the increase in blood lactate with no change in VO_2_ and measured biomechanical variables questions that lactate sampling from either leg or arm is an adequate method to establish arm or leg muscle activation during cross-country skiing. Furthermore, arm O_2_ extraction decreased during DP compared to DIA although both an increased pole force and muscle activation, pointing towards mechanical hindrance of blood flow during DP. Finally a greater ratio of upper- to lower-body muscle activation was associated with an attenuated arm O_2_ extraction in DIA, while increased cycle length, paralleled with increased recovery time and more active and dynamic lower body use increases O_2_ extraction especially in DP.

## Abbreviations

AEMG: Average electromyography
CaO_2_: O_2_ content of arterial blood
CfvO_2_: Oxygen content of venous femoral blood
CsvO_2_: Oxygen content of subclavian femoral blood
DIA: Diagonal stride
DIA_LIN_: Workload required achieving 90% of VO_2max_ during DIA
DP: Double poling
DP_LIN_: Workload required achieving 90% of VO_2peak_ during DP
EMG_RMS_: Root mean square electromyography
HR: Heart rate
HR_max_: Maximal heart rate
IEMG: Integrated electromyography
MVC: Maximal voluntary contraction
OBLA: Onset of blood lactate accumulation
SO_2_: Oxygen saturation
VCO_2_: Carbone dioxide production
V_E_: Minute ventilation
VO_2_: Oxygen uptake
VO_2max_: Maximal oxygen uptake
VO_2peak_: Peak oxygen uptake
